# Genome-Wide Comparative Analysis of Invertases in the *Salicaceae* with the Identification of Genes Involved in Catkin Fiber Initiation and Development

**DOI:** 10.3390/cimb47060423

**Published:** 2025-06-05

**Authors:** Hui Wang, Qianhua Tang, Jinyan Mao, Chang Jia, Zilu Qin, Yiqun Chen, Qingqing Liang, Xiaogang Dai, Yingnan Chen, Tongming Yin, Huaitong Wu

**Affiliations:** 1 State Key Laboratory of Tree Genetics and Breeding, Nanjing Forestry University, Nanjing 210037, China; zwh045687@njfu.edu.cn (H.W.); tangqianhua@njfu.edu.cn (Q.T.); maojinyan@njfu.edu.cn (J.M.); jiachang@njfu.edu.cn (C.J.); qinzilu@njfu.edu.cn (Z.Q.); 1599815146zz@gmail.com (Y.C.); liangqingqing@njfu.edu.cn (Q.L.); xgdai@njfu.edu.cn (X.D.); chenyingnan@njfu.edu.cn (Y.C.); tmyin@njfu.com.cn (T.Y.); 2 Co-Innovation Center for Sustainable Forestry in Southern China, Key Laboratory of Tree Genetics and Biotechnology of Educational Department of China, Key Laboratory of Tree Genetics and Silvicultural Sciences of Jiangsu Province, Nanjing Forestry University, Nanjing 210037, China

**Keywords:** *Populus deltoides*, *Salix suchowensis*, genome-wide, invertase, fluffy fibers

## Abstract

Invertase (INV) irreversibly converts sucrose to glucose and fructose during processes such as differentiation and organ development in plants, especially during the development of trichomes. Systematic identification and analysis of INVs in Salicaceae remain limited. Here, *INV* genes in *Populus deltoides* and *Salix suchowensis* were investigated, and their chromosomal localization, collinearity, gene structures, cis-regulatory elements, and phylogenetic relationships were comprehensively analyzed. Twenty and seventeen *INVs* were found, respectively, in *P. deltoides* and *S. suchowensis*, most of which were derived from a common ancestor and exhibited similar chromosomal distribution and high collinearity. Orthologs between the two species showed conservation of gene structures and promoter regulatory elements. Multi-species phylogenetic analysis identified an evolutionary clade associated with seed fiber development in *P. deltoides* and *S. suchowensis*. Further evaluation of *INV* expression in female catkins at various stages of seed fiber formation verified the predominance of *PdeVINV1*, *PdeVINV2*, *PdeVINV3*, and *PdeVINV4* in *P. deltoides*, as well as *SsuVINV1* and *SsuVINV2* in *S. suchowensis*, during critical phases of catkin fiber differentiation. These genes are likely to have significant regulatory roles in the initiation and development of catkin fiber cells. These findings provide a reference for future functional studies of INVs.

## 1. Introduction

Invertases (INVs) irreversibly convert sucrose to glucose and fructose, and are associated with processes such as carbon partitioning, metabolism, nutrient allocation, yield formation, signaling, and stress resistance [1,2]. Based on their solubility, subcellular localization, and pH preferences, INVs can be divided into two subfamilies, namely acid and neutral/alkaline INVs [3]. The former are further subdivided into cell wall-localized invertases (CWINVs) and vacuolar-soluble invertases (VINVs), while the latter are found in the cytoplasm and are termed neutral invertases (NINVs) [3]. CWINVs and VINVs share biochemical and molecular characteristics [4]. For example, both possess an N-terminal NDPD/NG motif and a WECPD catalytic domain, as described by the CAZy database for glycoside hydrolase family 32 [5,6,7]. Phylogenetic evidence suggests that CWINVs in higher plants evolved from VINVs ancestral to those in both higher and lower plants [8]. In contrast to CWINVs and VINVs, NINVs exhibit distinct structural and substrate-specific characteristics [9]. NINVs harbor two strongly conserved amino acid residues, Asp188 and Glu414, and are classified under glycoside hydrolase family 100 in the CAZy database [10,11]. Consequently, despite their common ability to hydrolyze sucrose, NINVs appear to utilize a mechanism that differs from that seen in CWINVs and VINVs. Moreover, CWINV and VINV sequences show greater diversity compared with those of NINVs, and the enzymes are also expressed in a wider range of tissues than NINVs [8]. This observation is consistent with findings from both plants and mammals, where genes with wide expression patterns tend to be structurally more conserved than those with narrower or specific tissue expression [12]. Collectively, these findings indicate stronger evolutionary conservation of NINVs than CWINVs and VINVs in higher plants.

INVs play diverse roles in plants. CWINVs can regulate sucrose allocation, influencing seed formation and pollen development [13,14]. The functions of VINVs in plants focus primarily on the development of fruit, together with responses to both biotic and abiotic stressors [10,11]. NINVs are associated with plant root development and growth, the maintenance of carbon balance in organelles, and responses to environmental stimuli [15,16]. VINVs are also important in initiating fiber cell formation. For instance, it was found that knockdown of *GhVIN1* in cotton blocked the formation of cotton fibers from ovule epidermal cells, likely by inhibiting genes involved in sugar pathways [17]. Other research has shown that the ability of VINVs to promote cotton fiber elongation is approximately 4–6 times that observed in roots, leaves, and stems, and remains at high levels during fiber elongation, reducing when elongation slows [18]. There are also studies showing that a *VINV* gene in *Populus trichocarpa* is indispensable for cellulose synthesis [19]. Recent research has revealed that sucrose degradation mediated by sucrose INV and sucrose synthase is critical for the development of poplar catkin fibers [20]. These findings are important in the investigation of INVs and their functions.

*Populus* and *Salix* are important tree species used in forestation and for timber in China as well as in other parts of the world, and they also have significant ecological, economic, and social value [21]. However, after pollination, female trees produce large numbers of catkin fibers that can cause allergies and other problems [22]. Catkin fibers result from the protrusion and differentiation of placental epidermal cells [23], in contrast to cotton fibers which develop from ovule epidermal cells [24], with both sharing similar differentiation and developmental processes. Previous studies in *Arabidopsis thaliana* have identified several genes controlling the initiation and early-stage morphogenesis of trichomes. Key regulators, including *GL1*, *TTG*, *GL3*, *GL2*, and *TTG2,* have been functionally characterized, demonstrating their roles in trichome initiation and developmental progression [25,26,27,28,29]. Additionally, *MYB5* and *MYB23* have been shown to modulate trichome elongation and branching patterns [30]. Beyond *Arabidopsis*, critical genes associated with fiber initiation in cotton have been identified and characterized over recent decades. The miR156 family regulates trichome distribution during floral development through binding to the promoter regions of *TCL1* and *TRY* [31]. MIXTA/MIXTA-like transcription factors, particularly *GhMYB25-like*, function as master regulators of cotton fiber initiation by orchestrating epidermal cell differentiation [32]. Distinct MBW complexes have been shown to interact with auxin signaling pathways to promote fiber initiation [33]. Several poplar genes associated with catkin fiber development have recently been identified. GST and LEA protein-encoding genes facilitate early seed trichome development via ROS-mediated pathways [34]. A functional interplay between PTOSUS2 and MYB transcription factors has been shown to be crucial for seed hair fiber development in poplar [20]. Comparative analyses in hairless soybean mutants have shown reduced expression of plant-specific *BURP* genes, providing evidence for their conserved role in seed trichome development across species [35]. Despite these advancements, current research is focused predominantly on Arabidopsis and cotton models, with relatively limited mechanistic understanding of fiber development in wind-pollinated species such as poplar and willow. Notably, a genome-wide identification of the *INV* gene family in *Populus trichocarpa* has laid foundational insights for further exploration of catkin fiber biosynthesis [36]. In the present study, *INV*s in *P. deltoides* and *S. suchowensis* were investigated, analyzing their chromosomal localization, collinearity, gene structure, and phylogenetic relationships. Analysis of temporal gene expression in catkin fibers at various developmental phases led to the identification of genes controlling fiber formation. The findings provide a valuable reference for addressing public health problems caused by catkin fibers and cultivating poplar and willow varieties without catkin fibers.

## 2. Materials and Methods

### 2.1. Identification and Analysis of INV Genes in P. deltoides and S. suchowensis

First, the genomic data of *Populus deltoides* (PRJNA598948) and *Salix suchowensis* (ASM1755242v1) analyzed in this study were retrieved from the NCBI database. A local database of the protein sequences within the genomes was constructed, and BLASTP was used to compare these sequences against those of *Populus tomentosa*, using an E-value cutoff of 0.01 [3]. Information on the INV protein domains Glyco_hydro_32N (PF00251), Glyco_hydro_32C (PF08244), and Glyco_hydro_100 (PF12899) was obtained from Pfam [37] and was used to screen for *P. deltoides* and *S. suchowensis* proteins containing the domains using HMMER 3.0 [38]. Candidate sequences obtained from both methods were merged and duplicates, incomplete sequences, or those lacking complete open reading frames (ORFs) were removed, leading to the identification of the INVs in the two species. The results were confirmed using the SMART database [18,39]. The chromosomal locations and protein lengths were determined from the BLAST-aligned sequences, and molecular weights (MWs) and theoretical isoelectric points (pIs) of the INV proteins were predicted using ProtParam [18].

### 2.2. Chromosomal Localization and Synteny Analysis

Using the INV IDs obtained in the previous step, information on chromosomal location in the two species was determined from their genome annotations using the “Gene Location Visualize from GTF/GFF” tool in TBtools (v1.09876), generating chromosomal distribution maps. The genome annotation and sequence files of both species were imported into the “One Step MCScanX” module in TBtools. The resulting collinearity, GFF, and ctl files were then loaded into the “Dual Synteny Plot” tool for visualization of the interspecies syntenic relationships [40] using the “Advanced Circos” module in TBtools (v1.09876). Finally, KaKs_Calculator 2.0 was utilized to compute synonymous (Ks) and nonsynonymous (Ka) substitution rates for the INV gene pairs.

### 2.3. Structural Characterization of INV Genes

The protein sequences of the INVs in both species were extracted using the “Fasta Extract” tool in TBtools (v1.09876) based on their respective protein files. A Newick-formatted phylogenetic tree file was generated from the protein sequences via the “One Step Build a ML Tree” module. Conserved motifs in the proteins were predicted with the “Simple MEME Wrapper” tool, resulting in a MEME.xml output file. The precise positions, numbers, and lengths of exons and introns within the predicted genes were determined. Finally, integration and visualization of the phylogenetic tree, conserved domains, and exon-intron structures were obtained using the “Gene Structure View” module in TBtools (v1.09876) with the generated tree, MEME.xml, and gene annotation files.

### 2.4. Phylogeny

Multiple sequence alignment of full-length INV proteins was conducted using ClustalX with default parameters. The alignment included sequences from two lower plants (*Physcomitrella patens* and *Selaginella moellendorffii*), two monocots (*Oryza sativa* and *Zea mays*), and seven dicots (*Populus deltoides*, *Salix suchowensis*, *Gossypium hirsutum*, *Arabidopsis thaliana*, *Solanum lycopersicum*, *Oryza sativa*, and *Solanum tuberosum*). The alignment was refined manually before phylogenetic tree construction [10]. A neighbor-joining (NJ) tree was constructed using MEGA 5.1 with Poisson substitutions, pairwise deletion, and 1000 bootstrap replicates [11]. Annotation and visualization were performed on the Interactive Tree of Life (iTOL) platform (https://itol.embl.de/upload.cgi/ accessed on 7 October 2024).

### 2.5. Collection and Processing of Plant Materials

The *P. deltoides* plant materials were collected from 15- to 20-year-old trees grown at Nanjing Forestry University, Nanjing City, Jiangsu Province, China. Female catkins of *Populus deltoides* were collected at nine time points: 3 days (−3D), 2 days (−2D), and 1 day (−1D) before pollination; the day of pollination (0D); and 1 day (1D), 2 days (2D), 3 days (3D), 5 days (5D), and 8 days (8D) after pollination. The *S. suchowensis* plant materials were collected at the Baima Experimental Base in Nanjing City, Jiangsu Province, China. Female catkins were collected at seven time points: 2 days before pollination (−2D); the day of pollination (0D); and 2 days (2D), 3 days (3D), 5 days (5D), 8 days (8D), and 15 days (15D) after pollination. All sampling followed a randomized design with three biological replicates. The plant materials were immediately frozen in liquid nitrogen and kept at −80 °C for subsequent RNA extraction.

### 2.6. Primers, RNA Extraction, and qRT-PCR

The coding sequences (CDS) of the *INV* genes in the two species were determined and primers were designed using Primer Premier 5. Primer specificities were verified via agarose gel electrophoresis (Supplementary Table S1) [41]. This procedure was conducted to prepare for subsequent analysis of *INV* expression. Total RNA was extracted from inflorescences using a Hibind Plant RNA Plus Kit (PORABIO, Hangzhou, China) and 1 μg of RNA from each sample was reverse-transcribed to cDNA using HisyGo RT Red SuperMix for qPCR (Vazyme, Nanjing, China). qRT-PCR was conducted on a 7500 Fast Real-Time PCR System (Applied Biosystems, Foster, CA, USA). The 20 μL reaction mixture included 1 μL each of the forward and reverse primers (10 μM), 1 μL of cDNA (diluted five-fold), 10 μL of 2× ChamQ Blue Universal SYBR qPCR Master Mix (Vazyme, Nanjing, China), and RNase-free water to make up the volume. The reaction involved initial denaturation at 95 °C for 15 s, followed by 40 cycles of 60 °C for 30 s, and 72 °C for 30 s. For *Populus deltoides*, the endogenous reference gene *PtUBQ* [42] was selected, while *ACT7* [43] served as the reference gene for *Salix suchowensis*. The stable expression of these validated reference genes was utilized as the normalization benchmark. Each biological sample was analyzed in triplicate, and a cross-sample average CT value normalization strategy was rigorously implemented to ensure comparability of gene expression levels across experimental groups [44].

### 2.7. Analysis of INV Cis-Acting Elements

Promoter sequences (2 kb upstream of the start codon) of the *INV* genes were obtained from the genome files of the two species. Cis-acting regulatory elements within the promoters were analyzed using the PlantCARE database [45].

## 3. Results and Discussion

### 3.1. Identification of INV Gene Families in P. deltoides and S. suchowensis

A total of 20 INVs were identified in *P. deltoides*, comprising 3 cell wall invertases (*PdeCWINV1–3*), 4 vacuolar invertases (*PdeVINV1–4*), and 13 cytosolic invertases (*PdeNINV1–13*). In *S. suchowensis*, 17 INV family members were detected, including 4 cell wall invertases (*SsuCWINV1–4*), 2 vacuolar invertases (*SsuVINV1–2*), and 11 cytosolic neutral invertases (*SsuNINV1–12*). The fundamental features of the acidic and alkaline/neutral INVs in both species are summarized in Table 1. For the Gene IDs of *P. deltoides* INV gene family members, the numbers from genome annotation files were used; for the Gene IDs of *S. suchowensis* INV gene family members, GeneBank accession numbers were used. The acidic INVs in *P. deltoides* had protein lengths of 460–661 amino acids (aa), pI values between 5.15 and 7.66, and MWs between 51,001.51 and 73,385.74 Da. In *S. suchowensis,* the acidic INVs ranged from 513 to 642 aa, with pI values of 5.21–8.97 and MWs of 57,757.56 to 72,151.16 Da. The alkaline/neutral INVs in *P. deltoides* were 543–722 aa in length, with pI values of 5.1–7.82 and MWs of 62,277.55 to 81,121.68 Da, while those in *S. suchowensis* ranged from 137 to 713 aa, with pI values of 5.18–7.68 and MWs of 15,469.01–80,109.69 Da (Table 1). Comparative analysis revealed distinct family expansions, such as in the acidic INV family, where *P. deltoides* contained one fewer cell wall INV but two additional vacuolar INVs compared to *S. suchowensis*.

In the alkaline/neutral INV family, *P. deltoides* was found to possess two additional cytosolic members compared with *S. suchowensis*. Overall, the *INV* gene family in *P. deltoides* contained three more members than that of *S. suchowensis*, which may reflect accelerated evolutionary rates in *S*. *suchowensis* leading to gene loss during divergence [46]. Variations in amino acid composition, MW, and pI among the family members suggest the functional diversification of these enzymes in specific microenvironments.

### 3.2. Chromosomal Distribution and Identification of Gene Duplication

**Figure 1:**
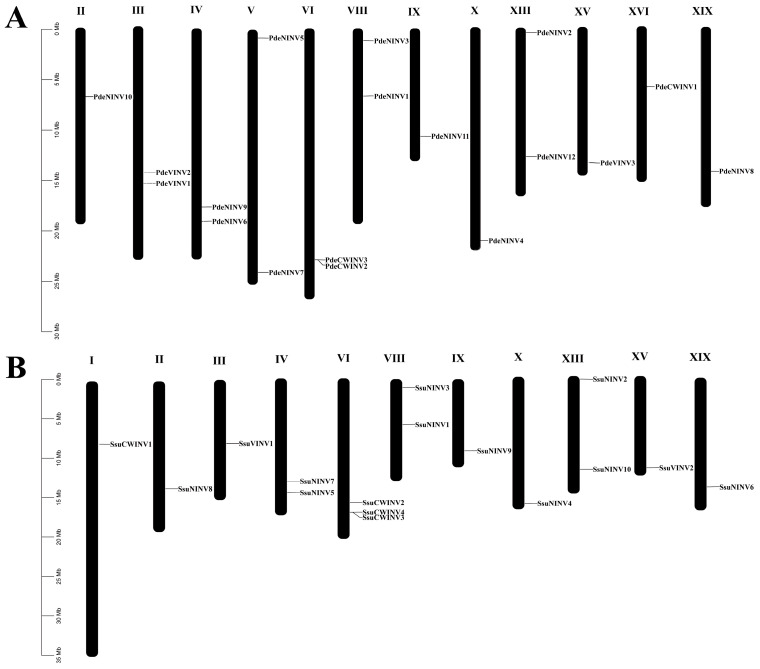
Chromosome maps of *INV* genes in the genomes of *P. deltoides* and *S. suchowensis*. (**A**) Eighteen *PdeINV* genes were mapped to 12 chromosomes in *P. deltoides*. (**B**) Sixteen *SsuINV* genes were mapped to 11 chromosomes in *S. suchowensis*. Genes showing tandem duplications are highlighted with red borders.

Nucleotide sequence alignments were performed with Tbtools (v1.09876), resulting in the mapping of the 18 *P. deltoides INV* genes to 12 chromosomes and the 16 *S. suchowensis INV* genes to 11 chromosomes (Figure 1). Two *P. deltoides* genes and one *S. suchowensis* gene could not be assigned to specific chromosomes but were localized to unassembled scaffold fragments, possibly the result of incomplete genome assemblies in these plant lineages.

The analysis revealed uneven chromosomal distributions of the *INV*s in both genomes. In *P. deltoides*, the maximum number of *INV* genes per chromosome was two, located on chromosomes 3, 4, 5, 6, 8, and 13 (Figure 1A). In contrast, chromosome 6 of *S. suchowensis* exhibited a relatively higher concentration with three *INV*s (Figure 1B). Notably, both genomes showed identical numbers of *INV*s at homologous positions on chromosomes 4, 6, 8, 9, 10, 13, 15, and 19, suggesting that these genes are orthologs inherited from a common ancestor [47]. Compared to *P. deltoides*, *S. suchowensis* lacked *INV* genes on chromosomes 5 and 16. The overall expansion of *INV*s in *P. deltoides* may have been driven primarily by the proliferation of neutral/alkaline invertases. The expansion of this subfamily may be associated with the conspicuous absence of an active phloem-loading process in this group of perennial woody plants [48]. A key observation was the presence of a tandemly duplicated *INV* gene pair on chromosome 6 in both *P. deltoides* and *S. suchowensis* (Figure 1).

**Figure 2:**
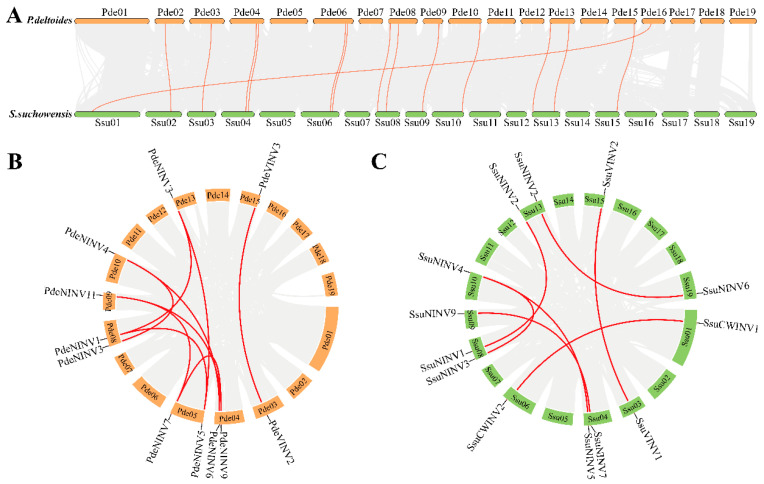
Collinearity analysis of *INV* genes. (**A**) Homologous relationships between *P. deltoides* and *S. suchowensis* on chromosomes. (**B**) Intraspecific collinear segments in *P. deltoides*. (**C**) Intraspecific collinear segments in *S. suchowensis*. Red lines indicate collinear *INV* gene pairs, and light-gray lines represent collinear segments.

Homologous relationships between the two species were analyzed, identifying 13 pairs of homologous genes (Supplementary Table S2). Eleven pairs of *INV* genes were located in the same chromosomal positions in the two species, while two pairs were in different positions. Among them, the linear relationship of one pair of genes could not be determined as they had not yet been mapped to chromosomes (Figure 2A). The other pair of homologous genes, *PdeCWINV1* and *SsuCWINV1*, were located on chromosome 16 of *Populus deltoides* and chromosome 1 of *Salix suchowensis*, respectively. This may be because poplars and willows originally shared a common ancestor. During evolution, they experienced a key salicinoid genome duplication event, which ultimately led to the differentiation into two major branches representing the current *Populus* and *Salix* genera [49]. Two main chromosomal rearrangements occurred during this process, with chromosome 1 of willow being a combination of poplar chromosome 16 and the lower part of chromosome 1, while willow chromosome 16 corresponds to the upper part of poplar chromosome 1 [46]. The results suggest that despite significant differences in distribution on some chromosomes, the overall distribution of *INV* genes was similar in the two genomes.

Duplications in gene families are driven primarily by both segmental and tandem duplications [50]. Single pairs of tandemly duplicated genes were observed in both species (*PdeCWINV2* and *PdeCWINV3*, *SsuCWINV3* and *SsuCWINV4*), with both pairs located on chromosome 6 (Figure 1). Nine pairs of segmentally duplicated genes involving 11 genes were identified in *P. deltoides* (Figure 2B), while seven pairs involving 12 genes were found in *S. suchowensis* (Figure 2C). The Ka/Ks values of these gene pairs were all below 1 (Supplementary Table S3), indicative of purifying selection, i.e., the removal of harmful variations in these gene pairs by natural selection during evolution. The results showed differences in the number of segmental gene duplications at the same positions in the two species, with two more duplications observed in *P. deltoides*. However, there were five events of segmental gene duplication at the same positions in both species, suggesting that *INV* genes may have undergone five duplication events in the common ancestry of poplars and willows. Following the divergence of the species, the willow genome showed markedly higher average substitution rates in the genes, together with a higher evolutionary rate. This suggests that willows have been subjected to more intense selection to remove harmful variations during evolution, resulting in the elimination of a greater number of genes. This may also be the reason why there are fewer *INV*s in *S. suchowensis* relative to *P. deltoides*.

### 3.3. Phylogenetic Relationships, Conserved Motifs, and Gene Structures of INV Genes in Populus and Salix

**Figure 3:**
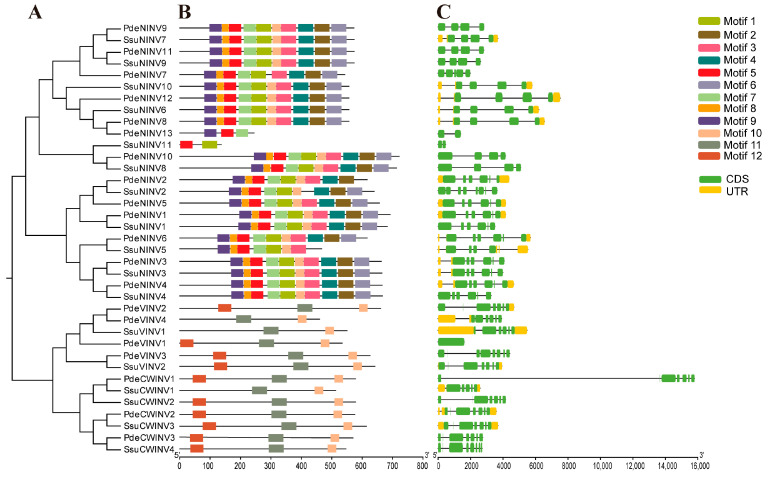
Phylogenetic relationships, conserved motifs, and gene structures of *INV*s in *P. deltoides* and *S. suchowensis*. (**A**) A phylogenetic tree constructed with 1000 replicates using the maximum-likelihood method in MEGA 5.1. (**B**) Conserved motifs in INV proteins. Individual motifs are represented by colored boxes, with solid black lines representing non-conserved sequences. The size and position of the motifs are shown according to the scale at the bottom of the figure. (**C**) Exon–intron structures of *INV* genes. Exons are represented in green, and introns are shown as solid black lines. The relative positions of introns and exons are shown according to the scale provided at the bottom of the figure.

The two species share a common ancestor, and subsequent evolution has led to both the loss and retention of gene homologs. A phylogenetic tree based on full-length INV protein sequences of the two species was constructed, with integration of conserved motif and gene structure information to provide a comprehensive analysis of similarities and divergences in the gene family. The tree contained two major clades, with one including neutral/alkaline INVs and the other acid INVs. The latter clade was divided further into CWINVs and VINVs (Figure 3A). In the CAZy database, neutral/alkaline INVs are classified within glycoside hydrolase family 100, characterized by the presence of Glyco_hydro_100 domains, while acid INVs belong to glycoside hydrolase family 32, characterized by Glyco_hydro_32N and Glyco_hydro_32C terminal domains.

The MEME program in TBtools identified 12 distinct conserved motifs (23–50 aa in length) in the INV proteins of both species, with 50% of the motifs being 50 residues in length (Supplementary Table S4). While the compositions of the motifs did not differ significantly between the two species, substantial differences were observed between the neutral/alkaline and acid INV clades. Neutral/alkaline INVs tended to share 10 conserved motifs (Motifs 1–10), with Motifs 1–9 being clade-specific. In contrast, acid INVs contained only three highly conserved motifs (Motifs 10–12). Motif conservation is typically associated with the tissue expression and function of genes. This would suggest that CWINVs and VINVs have similar functions in biological processes. Notably, *PdeNINV13* and *SsuNINV11* in the neutral/alkaline clade exhibited atypical patterns, containing Motifs 5/9/7 and Motifs 5/1, respectively. Within the acid INV clade, *PdeVINV4*, *SsuVINV1*, and *SsuCWINV1* lacked Motif 12 (Figure 3B).

Analysis of gene structures revealed substantial variability in exon–intron organization among the *INV* family members. *SsuNINV2*, *PdeVINV3*, and *SsuCWINV4* exhibited the most complex structures (eight exons and seven introns), while *PdeVINV1* had the simplest configuration. Statistical analysis showed that 5.4% (2/37) of *INV* genes contained one intron, while 91.89% (34/37) harbored more than one intron, and only 2.7% (1/37) lacked introns. Phylogenetic correlations demonstrated that most homologous gene pairs (such as *PdeNINV11* and *SsuNINV9*) within the same clade shared similar intronic patterns. However, minor structural divergences were observed in some paralogs, such as *PdeNINV9* (three introns) versus its homolog *SsuNINV7* (four introns) (Figure 3C).

### 3.4. Phylogenetic Analysis of INV Gene Families in 11 Plant Species

A total of 223 INV proteins from 11 plant species were used to investigate the evolutionary relationships among INVs in the plant kingdom. A phylogenetic tree was constructed using the neighbor-joining method in MEGA5.1 with 1000 bootstrap replicates. The eleven species included two lower plants (*Physcomitrella patens* and *Selaginella moellendorffii*), two monocots (*Oryza sativa* and *Zea mays*), and seven dicots (*Populus deltoides*, *Salix suchowensis*, *Gossypium hirsutum*, *Arabidopsis thaliana*, *Solanum lycopersicum*, *Oryza sativa*, and *Solanum tuberosum*) (Supplementary Table S5).

The phylogenetic tree revealed separation of the 223 INV proteins into two major groups corresponding to acid and alkaline/neutral INVs, respectively. The acid INVs formed four distinct clades (Clades I–IV), with Clades I and II belonging to the VINV subgroup, and Clades III and IV to the CWINV subgroup. Clade I included only members from the two lower plant species, while Clade II consisted solely of proteins from higher angiosperms. Notably, four VINV proteins from *P. deltoides* were observed in Clade II, while *S. suchowensis* contained only two. Clade III members were all derived from dicots, with both *P. deltoides* and *S. suchowensis* possessing two CWINV genes each. Clade IV included proteins from both monocots and dicots, with *P. deltoides* containing one CWINV protein and *S. suchowensis* two. No proteins from lower plants were observed in Clades III or IV. The alkaline/neutral invertases (NINVs) formed two separate clades, namely Clades V and VI. Clade V comprised five *S. suchowensis* NINVs and six *P. deltoides* NINVs, while Clade VI contained six *S. suchowensis* and seven *P. deltoides* NINV proteins. *P. deltoides* had one additional protein in each clade compared to *S. suchowensis*. Notably, most proteins from *P. deltoides*, *S. suchowensis*, and *Gossypium hirsutum* clustered within shared subclades.

**Figure 4:**
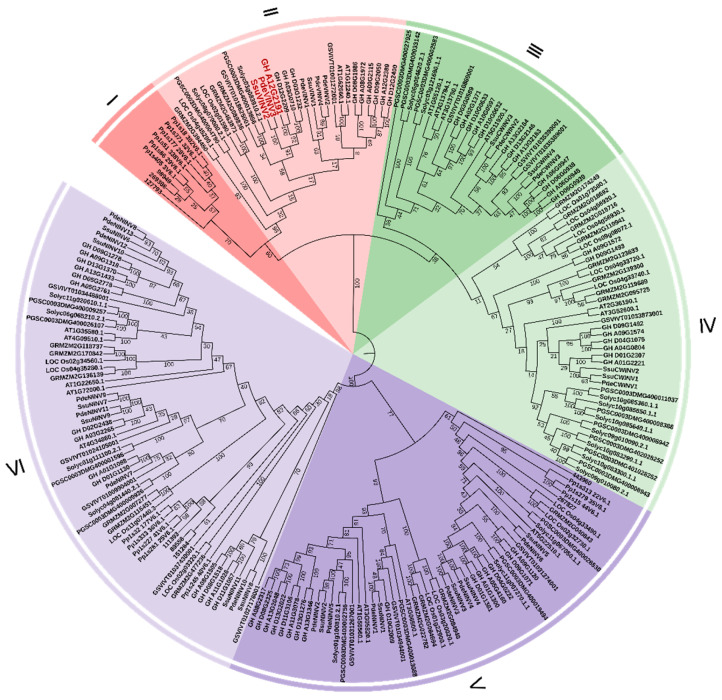
Phylogenetic relationships of INVs in 11 plant species. The neighbor-joining tree was constructed in MEGA 5.1 with 1000 bootstrap replicates. All INVs were clustered into six different groups (I–Ⅵ), represented by different colors. Groups Ⅰ and Ⅱ in the red area represent VINVs, Groups Ⅲ and Ⅳ in the green area are CWINVs, and Groups Ⅴ and Ⅵ in the purple area are NINVs. The 11 plant species are *Physcomitrella patens*, *Selaginella moellendorffii*, *Oryza sativa*, *Zea mays*, *Populus deltoides*, *Salix suchowensis*, *Gossypium hirsutum*, *Arabidopsis thaliana*, *Solanum lycopersicum*, *Oryza sativa*, and *Solanum tuberosum*.

An analysis of the diversification of the three types of invertases, CWINVs, VINVs, and NINVs, during their evolution from lower plants to higher plants revealed that VINVs from both lower plant types were clustered in Clade I, while VINVs from higher plants formed an independent cluster within Clade II. This suggests that VINVs may have diversified more rapidly due to specific functional adaptations during evolution, leading to significant differences in their sequences. CWINVs were not detected in lower plants, suggesting that CWINVs in higher plants may have originated from a common ancestor shared with VINVs in lower plants, and their functional differentiation may occurred as adaptations to vascular system developmental requirements. NINVs from both lower and higher plants together formed two tightly associated clades, reflecting stronger functional or structural constraints during evolution, and resulting in markedly greater conservation compared with other invertases [8]. Analyses of functional conservation have indicated that genes within the same taxon tend to have similar or identical functions, enabling prediction of the functions of related genes [51]. A previous study reported that silencing of *GhVIN1* (Gene ID: GH_A12G2191) in cotton could block fiber initiation from the ovule epidermis [17]. Poplar and willow catkin fibers are similar to cotton fibers in terms of tissue origin and function, suggesting that they may share similar regulatory mechanisms. The cotton gene *GhVIN1* was located in Clade II. The *P. deltoides* INVs in Clade II included *PdeVINV1*, *PdeVINV2*, *PdeVINV3*, and *PdeVINV4*, while the INVs in *S. suchowensis* were *SsuVINV1* and *SsuVINV2*. Among them, *PdeVINV3* and *SsuVINV2* were found to be present in the same subclade as the cotton INV (Figure 4). It is thus speculated that these genes may be associated with the development of seed flocculent fibers.

### 3.5. Identification of INVs Associated with the Development of Poplar and Willow Floss

**Figure 5:**
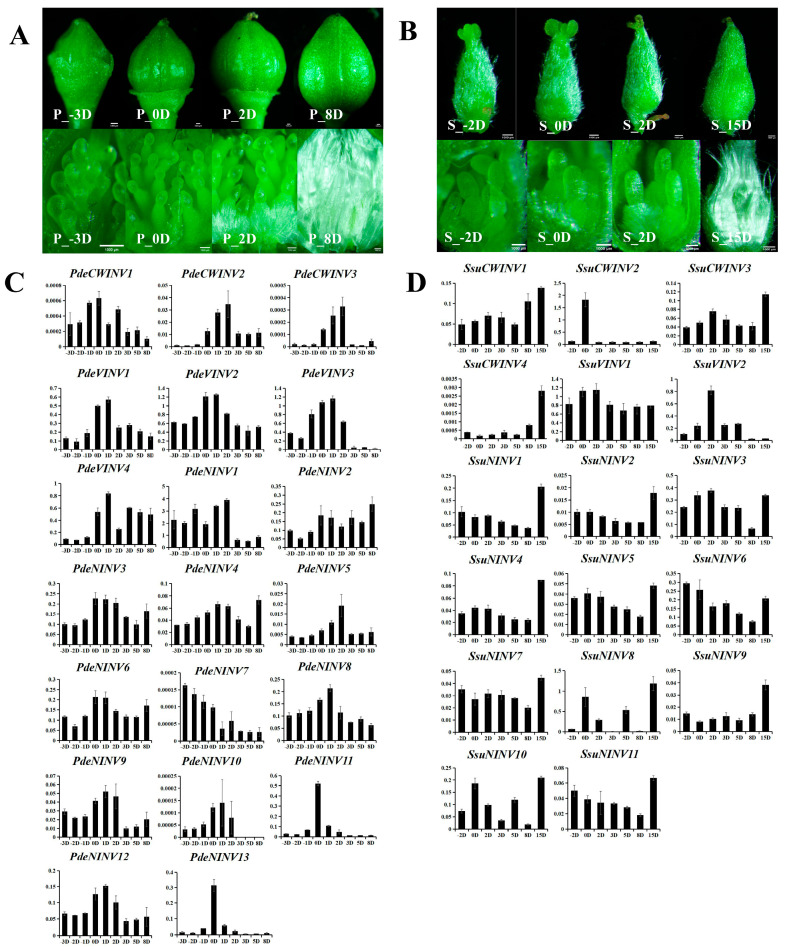
Expression of *INV* genes in *P. deltoides* and *S. suchowensis* during seed fiber initiation and development. (**A**) Phenotypes of seed hairs and the development of internal seed fibers in *P. deltoides* at different stages. (**B**) Phenotypes of seed hairs and the development of internal seed fibers in *S. suchowensis* at different stages. Scale bar = 1 mm in both (**A**,**B**). (**C**) Expression of *INV* genes in *P. deltoides*. (**D**) Expression of *INV* genes in *S. suchowensis*. The *x*-axis represents different periods and the *y*-axis represents relative expression levels. The values shown are the mean ± standard deviation of three biological replicates (*p* < 0.01, two-tailed Student’s *t*-test).

Fibers are flocculent fibers, produced by female poplars and willows after maturation of the fruit, that aid seed dispersal. Their formation results from the differentiation of placental epidermal cells. To examine the regulatory effects of INVs on catkin fibers, female inflorescences were collected at different stages and the expression of *INV* genes was analyzed. Consistent with previous reports [17,20], the cells producing catkin fibers began to differentiate from placental cells 1–2 days after pollination (Figure 5A,B). Therefore, the day of pollination was taken as day 0 (0D). In poplar, female inflorescences were collected at 9 time points, namely 3 days (−3D), 2 days (−2D), and 1 day (−1D) before pollination; the day of pollination (0D); and 1 day (1D), 2 days (2D), 3 days (3D), 5 days (5D), and 8 days (8D) after pollination. In willow, female inflorescences were collected at seven time points, namely 2 days before pollination (−2D); the day of pollination (0D); and 2 days (2D), 3 days (3D), 5 days (5D), 8 days (8D), and 15 days (15D) after pollination. qRT-PCR was used to analyze the expression patterns of the *INV*s. It was speculated that INVs, which are preferentially expressed during the initial differentiation of catkin fibers, may be key to the regulation of fiber development.

The results showed the presence of obvious flocculent fibers on the surfaces of the placental cells in female inflorescences from *P. deltoides* at 2D, with complete enclosure of the ovules at 8D. Similarly, obvious flocculent fibers were also apparent on the placental cell surfaces of female inflorescences from *S. suchowensis* at 2D, with complete enclosure of the ovules at 15D (Figure 5A,B). It is thus speculated that the critical period for the initiation of the catkin-like fibers in *P. deltoides* and *S. suchowensis* is within 0–2 days (0–2D). Analysis of gene expression showed that all *INV* genes were expressed during catkin fiber development. Among them, *PdeVINV1*, *PdeVINV2*, *PdeVINV3*, and *PdeVINV4* were highly expressed in poplar at the start of fiber differentiation (0–2D), as were *SsuCWINV2*, *SsuVINV1*, and *SsuVINV2* in willow (0–2D) (Figure 5C,D). These genes all belonged to the VINV subclass. The expression of these genes increased continuously during the development of catkin fibers in both species, with peak levels at the start of fiber development (0–2D), and decreased significantly upon fiber maturation. In contrast, both *CWINV* and *NINV* genes showed low overall expression throughout the catkin fiber development cycle. Although several individual genes (such as *SsuCWINV2*) exhibited high expression levels, there was no trend of upregulation during the critical period of catkin fiber initiation. The results suggest that VINVs are more closely involved in the initiation and development of catkin fibers compared to cell wall and cytoplasmic INVs, which is consistent with the earlier findings of Yang X et al. [20]. The phylogenetic analysis of INVs from multiple species showed that *PdeVINV3* and *SsuVINV2* were located on the same branch as *GhVIN1* that regulates cotton fiber formation, implying that *PdeVINV3* and *SsuVINV2* may be key regulators of catkin fiber formation in poplar and willow, respectively. The analysis of gene expression profiles thus clarified the key roles of VINVs in the initiation and development of catkin fibers in poplar and willow.

### 3.6. Promoter Element Analysis

To elucidate the regulatory elements of *INVs*, cis-acting elements in the promoter regions of 37 *INVs* from the two species were analyzed. The results demonstrated that these cis-acting elements could be classified into 12 categories (Supplementary Table S6). Previous studies have indicated that phytohormone-responsive elements, including elements associated with responses to gibberellin, abscisic acid, and auxin, can induce expression of *CWINV* genes. The presence of these phytohormone-related regulatory elements was observed in VINVs and NINVs from both species. In contrast, elements responding to abiotic stressors, such as light and low temperature, as well as MYB-binding sites linked to drought responses, have been found to regulate the expression of most VINVs and NINVs [49]. Notably, gibberellin-responsive elements have been shown to alleviate the glucose-mediated suppression of CWINVs, thereby modulating intracellular glucose metabolism and maintaining normal cellular growth in fruit [23]. Furthermore, INV-mediated hexose signaling can act upstream of MYB transcription factors and auxin signals, serving as an essential component for fiber initiation [13].

**Figure 6:**
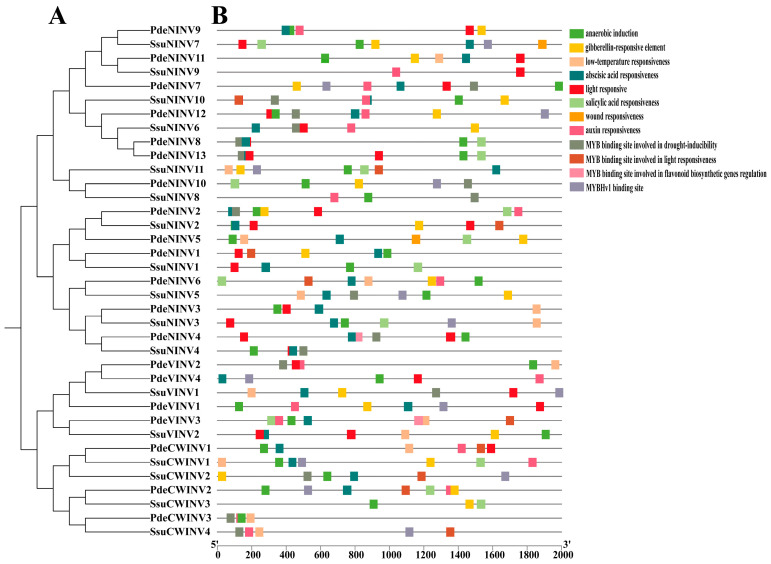
(**A**) Phylogenetic relationships. (**B**) Cis-acting elements.

Here, it was found that cis-acting elements involved in abscisic acid responsiveness were abundant among INVs in both poplar and willow, with 30 members harboring such elements. Conversely, elements associated with responses to wounding were the least common, identified only in *PdeNINV5*. A total of 17 genes contained auxin responsiveness-related elements, while 22 included MYB-binding elements and 14 exhibited MYBHv1 binding site-related elements. *PdeVINV3*, located in the same clade as cotton *GhVIN1*, was observed to contain two MYB transcription factor-associated regulatory elements and one auxin-responsive element. In contrast, *SsuVINV2*, while lacking MYB and auxin-responsive elements, harbored two hormone-related response elements associated with responses to abscisic acid and anaerobic induction, respectively. It is hypothesized that *SsuVINV2* regulates seed fiber initiation and development through these hormone-responsive elements. Together with these three genes in the major subclass II clade, the poplar genes *PdeVINV1*, *PdeVINV2*, and *PdeVINV4* were each found to possess one MYB transcription factor-associated regulatory element and one auxin-responsive element, while *SsuVINV1* contained two MYB regulatory elements. Based on these findings, it is proposed that these genes regulate seed fiber initiation and development, potentially mediated by interactions with MYB transcription factors and auxin-associated pathways (Figure 6B).

## 4. Conclusions

This study identified 20 and 17 *INV*s in poplar and willow, respectively. Chromosomal distribution, gene structures, sequence conservation, and gene duplications associated with these genes were comprehensively analyzed. The results indicated that INVs in poplar and willow were inherited from a common ancestor, with several family members undergoing further species-specific evolution. Phylogenetic analyses indicated that proteins on several branches of the tree were associated with the regulation of seed fiber development. Analysis of *INV* expression patterns during female flower development identified *PdeVINV3* and *SsuVINV2* as key regulators of catkin fiber formation. These findings provide references both for further investigation into INV functions in poplar and willow and for the study of the molecular regulation of catkin fiber development.

## Figures and Tables

**Table 1:** Physicochemical properties of the *INV* gene family in *P. deltoides* and *S. suchowensis*.

Gene Symbol	Gene ID	Chr	Start	End	Amino Acid	PI	Mw
*PdeCWINV1*	EVM0014612	chr16	5,978,219	5,994,019	578	65,117.66	6.58
*PdeCWINV2*	EVM0008374	chr6	22,923,121	22,926,712	576	65,562.07	5.15
*PdeCWINV3*	EVM0008949	chr6	22,919,482	22,922,237	570	64,803.28	7.66
*PdeVINV1*	EVM0021361	chr3	15,565,345	15,566,949	534	59,632.07	5.3
*PdeVINV2*	EVM0032360	chr3	14,483,232	14,487,904	661	73,385.74	5.92
*PdeVINV3*	EVM0005782	chr15	13,459,417	13,463,828	626	70,134.85	5.3
*PdeVINV4*	EVM0034321	Contig00422	29,267	33,178	460	51,001.51	5.36
*SsuCWINV1*	KAG5253080	chr1	7,929,888	7,932,471	513	57,757.56	8.66
*SsuCWINV2*	KAG5243975	chr6	15,728,185	15,732,344	578	65,683.72	8.97
*SsuCWINV3*	KAG5244133	chr6	16,948,561	16,952,254	614	69,846.19	5.7
*SsuCWINV4*	KAG5244132	chr6	16,945,043	16,947,752	547	62,500.56	6.68
*SsuVINV1*	KAG5249197	chr3	8,030,054	8,035,528	550	61,029.28	5.92
*SsuVINV2*	KAG5230526	chr15	11,591,356	11,595,290	642	72,151.16	5.21
*PdeNINV1*	EVM0016105	chr8	6,703,225	6,707,387	692	77,974.79	6.1
*PdeNINV2*	EVM0028453	chr13	472,802	477,178	617	69,982.91	5.83
*PdeNINV3*	EVM0036036	chr8	1,215,796	1,219,873	663	74,292.93	6.45
*PdeNINV4*	EVM0037445	chr10	21,183,172	21,187,826	666	74,536.12	6.02
*PdeNINV5*	EVM0003860	chr5	773,232	777,399	657	74,839.51	5.78
*PdeNINV6*	EVM0006001	chr4	19,121,245	19,126,930	617	69,484.1	7.82
*PdeNINV7*	EVM0018844	chr5	24,044,123	24,046,098	543	62,277.55	6.01
*PdeNINV8*	EVM0008004	chr19	14,355,560	14,362,118	557	63,338.65	5.92
*PdeNINV9*	EVM0037237	chr4	17,702,452	17,705,280	573	65,529.25	6.11
*PdeNINV10*	EVM0031867	chr2	6,833,729	6,837,875	722	81,121.68	5.1
*PdeNINV11*	EVM0022732	chr9	10,672,157	10,674,968	574	65,655.41	5.92
*PdeNINV12*	EVM0014961	chr13	12,780,570	12,788,090	557	63,568.04	6.35
*PdeNINV13*	EVM0012606	Contig01214	1913	3300	245	27,475.09	5.99
*SsuNINV1*	KAG5240243	chr8	5,743,869	5,747,374	683	77,291.94	6.14
*SsuNINV2*	KAG5232299	chr13	345,772	349,410	640	72,308.86	5.97
*SsuNINV3*	KAG5239587	chr8	1,071,815	1,075,805	665	74,395.89	6.02
*SsuNINV4*	KAG5237853	chr10	15,999,564	16,002,831	667	74,351.93	5.87
*SsuNINV5*	KAG5248034	chr4	14,455,452	14,460,986	467	52,028.75	5.37
*SsuNINV6*	KAG5224657	chr19	13,831,154	13,837,354	557	63,446.95	6.23
*SsuNINV7*	KAG5247877	chr4	13,017,770	13,021,450	574	65,729.31	6.12
*SsuNINV8*	KAG5251679	chr2	13,585,135	13,590,231	713	80,109.69	5.18
*SsuNINV9*	KAG5239072	chr9	9,074,707	9,077,316	574	65,572.23	6.14
*SsuNINV10*	KAG5233215	chr13	11,775,507	11,781,303	557	63,485.93	6.39
*SsuNINV11*	KAG5251679	Contig02329	58,959	59,417	137	15,469.01	7.68

## Data Availability

Data are contained within the article or Supplementary Materials.

## Supplementary Materials

The following supporting information can be downloaded at https://www.mdpi.com/article/10.3390/cimb47060423/s1.

## Abbreviations

The following abbreviations are used in this manuscript:

MDPI	Multidisciplinary Digital Publishing Institute
DOAJ	Directory of open access journals
TLA	Three-letter acronym
